# Initiating a participatory action research process in the Agincourt health and socio–demographic surveillance site

**DOI:** 10.7189/jogh.07.010413

**Published:** 2017-06

**Authors:** Oghenebrume Wariri, Lucia D’Ambruoso, Rhian Twine, Sizzy Ngobeni, Maria van der Merwe, Barry Spies, Kathleen Kahn, Stephen Tollman, Ryan G Wagner, Peter Byass

**Affiliations:** 1 Centre for Global Development and Institute of Applied Health Sciences, University of Aberdeen, Aberdeen, Scotland, UK; 2 Department of Paediatrics, Federal Teaching Hospital Gombe, Gombe, Nigeria; 3 Umeå Centre for Global Health Research, Division of Epidemiology and Global Health, Department of Public Health and Clinical Medicine, Umeå University, Umeå, Sweden; 4 MRC/Wits Rural Public Health and Health Transitions Research Unit (Agincourt), School of Public Health, Faculty of Health Sciences, University of the Witwatersrand, Johannesburg, South Africa; 5 INDEPTH, Accra, Ghana; 6 Directorate for Maternal, Child, Women and Youth Health and Nutrition, Mpumalanga Department of Health, South Africa

## Abstract

**Background:**

Despite progressive health policy, disease burdens in South Africa remain patterned by deeply entrenched social inequalities. Accounting for the relationships between context, health and risk can provide important information for equitable service delivery. The aims of the research were to initiate a participatory research process with communities in a low income setting and produce evidence of practical relevance.

**Methods:**

We initiated a participatory action research (PAR) process in the Agincourt health and socio–demographic surveillance site (HDSS) in rural north–east South Africa. Three village–based discussion groups were convened and consulted about conditions to examine, one of which was under–5 mortality. A series of discussions followed in which routine HDSS data were presented and participants’ subjective perspectives were elicited and systematized into collective forms of knowledge using ranking, diagramming and participatory photography. The process concluded with a priority setting exercise. Visual and narrative data were thematically analyzed to complement the participants’ analysis.

**Results:**

A range of social and structural root causes of under–5 mortality were identified: poverty, unemployment, inadequate housing, unsafe environments and shortages of clean water. Despite these constraints, single mothers were often viewed as negligent. A series of mid–level contributory factors in clinics were also identified: overcrowding, poor staffing, delays in treatment and shortages of medications. In a similar sense, pronounced blame and negativity were directed toward clinic nurses in spite of the systems constraints identified. Actions to address these issues were prioritized as: expanding clinics, improving accountability and responsiveness of health workers, improving employment, providing clean water, and expanding community engagement for health promotion.

**Conclusions:**

We initiated a PAR process to gain local knowledge and prioritize actions. The process was acceptable to those involved, and there was willingness and commitment to continue. The study provided a basis from which to gain support to develop fuller forms of participatory research in this setting. The next steps are to build deeper involvement of participants in the process, expand to include the perspectives of those most marginalized, and engage in the health system at different levels to move toward an ongoing process of action and learning from action.

Estimates suggest that over 1 billion people, the majority from low and middle–income countries (LMICs), experience barriers to access of good quality health care [1]. The problem can be related to a lack of information about the needs of those who are excluded from access. Health policy and planning that does not account for those who are excluded can give rise to a self–sustaining situation in which the health system, albeit inadvertently, is organized to maintain their exclusion [2–4]. The first step toward overcoming this situation requires reliable evidence about those who are excluded in order to inform the equitable organization of care [5–7].

In the absence of complete vital health data, pragmatic alternatives such as Verbal Autopsy (VA) has become an important source of information on population health. VA is a survey based method frequently used to investigate deaths identified as part of the routine operations of health and demographic surveillance sites (HDSSs). In a VA, final carers of deceased persons are interviewed about their relatives’ terminal symptoms using a standard, validated questionnaire [8]. Data are then interpreted to determine probable medical causes of death [8]. Approximately 48 million deaths are unregistered worldwide, three–quarters of which occur in LMICs [7]. In this context, VA has become a critical source of information for vital statistics and health systems strengthening [4,9–11].

Avoidable mortality among disadvantaged groups is strongly influenced by social conditions. Information on how the social determinants of health inequalities influence access to health services and health outcomes is therefore necessary to prioritize equity in health policy and planning [12]. An extension of this school of thought prioritizes participatory research as an approach to elicit information on the social determinants of health inequalities by enabling the perspectives of disadvantaged populations.

Participation is a broad term encompassing a range of interpretations from non– and marginal participation to fuller forms concerned with power and empowerment [13,14] (**Figure 1**). Narrower forms of participation are characterized by activities such as information sharing and consultation, considering participation as a means to an end in which: “donors or governments [use] community resources (land, labour, money) to offset the costs of providing services” [15].

**Figure 1:**
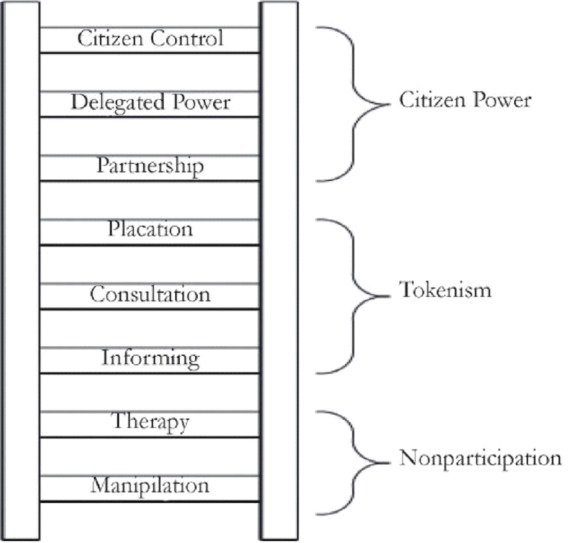
Ladder of citizen participation [13].

Broader views of participation consider it as an end in itself, where communities own the process and its development, where: “local communities [take] responsibility for diagnosing and working to solve their own health and development problems” [15]. In this scenario, active participation is related to community control and empowerment [13]. Here, the process aims to redresses power and information asymmetries between communities and the political and administrative forces that shape health policies [16,17].

Participatory action research (PAR) is an approach concerned with fuller forms of participation. In PAR, knowledge is co–created, acted on, and learning from action is sought to bring about and sustain change [17]. PAR methods change the usual way of doing research that emphasizes a divide between the researchers and the researched, transforming the subjects of research toward roles as active researchers and agents of change [18].

The research was conducted in a rural province of South Africa. Described as one of the most unequal societies in the world, the South African health system faces a complex ‘quadruple’ burden of socially patterned mortality comprising: chronic infectious diseases (characterized by HIV/AIDS and tuberculosis), non–communicable conditions, maternal and child mortality, and mortality owing to injury and violence [19]. The burden of HIV is high and highly unequal. Prevalence in black populations is 40–50 times that of white and in adolescents, risks are eight times higher in females than males [20].

Despite entrenched inequalities, the post–apartheid policy context in South Africa is progressive and inclusive. There is a constitutional commitment to the right to health and community participation for Primary Health Care (PHC) [21], and in 2011 National Health Insurance (NHI) was launched as a bold commitment to Universal Health Coverage (UHC) [22–24]. Significant gaps exist between policy and implementation however, in a system characterized by chronic underinvestment, a human resource crises, widespread corruption, poor stewardship and deteriorating infrastructure [25].

## Aims and objectives

Robust evidence on context, health and risk for groups excluded from health and information systems is crucial to inform equitable health systems responses. The overall aims of the research were to initiate a PAR process with communities in a health and socio–demographic surveillance site (HDSS) and produce evidence of practical relevance. The objectives were to engage with communities to examine VA data from HDSS, develop local knowledge around the VA data, and set priorities for local services.

## METHODS

### Study setting

**Figure 2:**
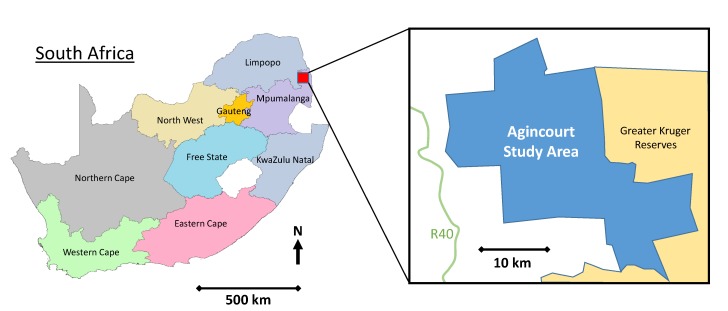
Map of Agincourt HDSS in rural northeast South Africa.

The study was conducted at the MRC/Wits Rural Public Health and Health Transitions Research Unit, which oversees the Agincourt HDSS located in rural Mpumalanga, a province of 4 million people in rural northeast South Africa. Established in 1992, the HDSS covers a population of approximately 115 000 people, over 450km^2^, 31 villages, and 20 000 households [26,27] (**Figure 2**). A dedicated Public Engagement Office works to enhance community and health systems engagement at different levels. The office regularly provides data and discusses research findings with the community and health system at different levels.

Serving the Agincourt study area within a radius of 20–60 km, is a network of ten government run PHC clinics that provide free basic outpatient health services during regular working hours. Services include routine maternal and child health interventions (including integrated management of childhood illnesses, well child visits, growth monitoring, routine immunizations), sexual and reproductive health services, testing and treatment for sexually transmitted infections, including HIV, minor trauma and routine care for chronic illnesses [28]. In 2015, attendance at antenatal care (ANC) clinics before 20 weeks of pregnancy in Mpumalanga was 56%, 80% of children one year and below had complete immunization, and the facility–based under–5 mortality rate was 8.3% against a target of 5% [29]. There are also limited private health care services in the area.

### Initiating PAR

**Figure 3:**
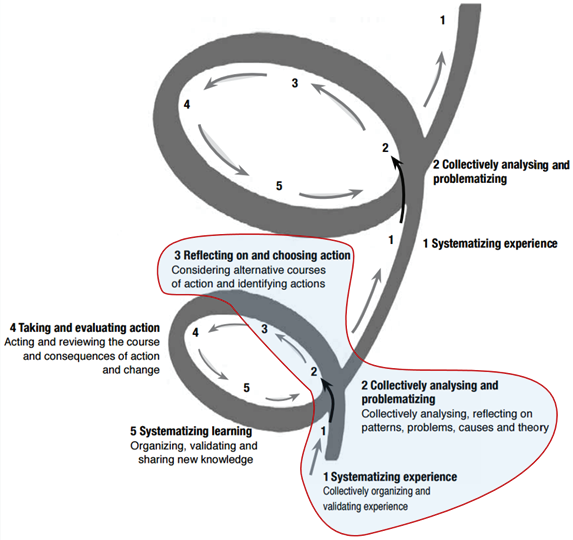
PAR process, with the initial elements highlighted [30].

We developed a process based on PAR. PAR is a non–linear, context specific process, with cycles of observing, reflecting, acting and learning from action. The repeated cycles build a sustained process that enables community ownership [30]. Within the time and resources available, it was possible to initiate the process and so the following description offered by Loewenson et al was adopted: “start by obtaining an insight into the communities and their conditions. This provides the information to support inclusion in the work, to systematize experience and to draw out priorities for attention” [30] (**Figure 3**).

We defined communities geographically, as residents of a specific area with shared social and health conditions. To prioritize and maintain prior linkages, we attempted to re–engage participants involved in a previous community–based participatory research (CBPR) pilot study in the Agincourt HDSS [31]. In the previous study, three village–based discussion groups had been convened. Villages had been selected on the basis of demographic variation and feasibility (**Table 1**) and in each village, discussion groups comprised women of reproductive age, family members, traditional healers, religious leaders, community health volunteers, health workers and community leaders. To mitigate any potential biases due to power differentials, in one village, the group consisted of women only (**Table 2**).

**Table 1:** Characteristics of selected villages

	Village–-based discussion group		
	**A**	**B**	**C**
Number of households	1178	932	647
Population, total	6158	4827	3705
Population, male	3005	2305	1781
Population, female	3147	2522	1924
Population, children under 5	647	513	458
Population, children of school age	1911	1410	1167

Source: [32].

**Table 2:** Composition of village based discussion groups

Participants*	Group			Total
**A**	**B**	**C**
Women of reproductive age (WRA)	1	1	2	4
Family members†	2	2	2	6
Traditional healers	1	1	2	4
Religious leaders and elders	1	2	2	4
Community health volunteers‡	1	1		2
Community/village officials‡	1	1		2
Community/village health workers‡	1	1		2
Total	8	8	8	24

*All participants recruited were 18 years of age or older. Although participants are likely to be categorized by more than one role in the community, one role per individual was considered for the purposes of convening the focus groups. We agreed roles with participants to identify what they feel to be their primary role in the community.

†Close relative: parents, grandparents, siblings, children, in–laws, nieces, nephews and cousins.

‡We acknowledged that people with working arrangements, particularly village health workers and village officials may not be available for a series of six weekly meetings. We also acknowledged the ethical imperative of engaging participants who would otherwise be involved in earning income and or the provision of public services. The groups were therefore based on these compositions, with careful consideration of minimizing disruption to local services.

Public Engagement Office staff approached individuals involved in the earlier CBPR study in villages, and described the current study, activities and intended outputs. Written consent forms and information sheets were provided, and participants were invited to ask questions at the time, or afterwards by telephone. For those willing to be involved, a convenient time was arranged for the first meeting at which participants were asked to sign and return the consent forms. Through this process, all participants from the prior study agreed to be involved.

### Data collection

In the first meeting, and to encourage participant control over how the topics for discussion were framed, we asked people’s opinions about conditions to examine. We also consulted the Directorate for Maternal Child, Women and Youth Health and Nutrition (MCWYH&N) in the provincial Department of Health (including co–authors BS and MVDM) and considered conditions with high prevalence rates identified in Agincourt HDSS. Through this approach, under–5 mortality and HIV–related mortality were selected. The discussion groups then embarked on a series of six weekly meetings to consider the conditions in terms of causes, contributory factors, and actions to address the identified issues (**Table 3**). This paper reports on the process as it related to under–5 mortality, the results on HIV–related mortality are reported elsewhere [33].

**Table 3:** Schedule of village–based meetings

	Week 1	Week 2	Week 3	Week 4	Week 5	Week 6
		Under–5 mortality		HIV–related mortality		
	Introduction and recruitment	Life histories and collective analysis	Collective analysis (continued) and action agendas	Life histories and collective analysis	Collective analysis (continued) and action agendas	Preliminary feedback and reflections on process
**Group:**						
A	A, 1	A, 2	A, 3	A, 4	A, 5	B, 6
B	B, 1	B, 2	B, 3	B, 5	B, 5	B, 6
C	C, 1	C, 2	C, 3	C, 5	C, 5	C, 6
Total number of meetings						18

### Subjective perspectives: VA data and life histories

In the second meetings, VA data on under–5 deaths were presented. 110 such deaths had been recorded by Agincourt HDSS in 2012 and 2013. The leading causes of death were acute respiratory infection (including pneumonia), HIV/AIDs–related death and malaria accounting for 18%, 15% and 13% of the total burden respectively. Overall, 61% of deaths were due to infectious causes. Furthermore, 49% of deaths occurred among children 1–4 years of age, 30% to infants and 21% to neonates (**Table 4**). The VA data also contained indicators on the circumstances of mortality, developed in the same project [35]. These data indicated multiple problems with access to care at and around the time of death. Specific issues identified were: families not calling for help (34% of all problems reported), not going to a facility at the time of death (29% of all problems reported), and that the overall costs of care were unaffordable (14% of all problems reported) (**Table 5**).

**Table 4:** Cause–specific mortality fraction (CSMF): all under–5 deaths, age/sex sub–groups

Cause of death	Age group			Sex		
	**Neonate (<28 days)**	**Infant (1–11 months)**	**Under 5 (1–4 years)**	**Female**	**Male**	**n (%)**
						
**Infectious:**		**27**	**40**	**38**	**29**	**67 (61)**
Acute respiratory infection including pneumonia		9	11	15	5	20 (18)
HIV/AIDS related death		2	14	7	9	16 (15)
Malaria		6	8	5	9	14 (13)
Diarrheal diseases		8	5	9	4	13 (12)
Meningitis and encephalitis		1	1	1	1	2 (2)
Pulmonary tuberculosis			1	1		1 (1)
Other and unspecified infectious disease		1			1	1 (1)
Neonatal:*	**16**	**2**	**1**	**9**	**10**	**19 (17)**
Neonatal pneumonia	7			6	1	7 (6)
Congenital malformation	1	2	1	1	3	4 (4)
Prematurity	3			1	2	3 (3)
Birth asphyxia	3			1	2	3 (3)
Neonatal sepsis	1				1	1 (1)
Other and unspecified neonatal cause of death	1				1	1 (1)
**Indeterminate**	**7**		**2**	**6**	**3**	**9 (8)**
**External:**		**2**	**6**	**3**	**5**	**8 (7)**
Accidental drowning/submersion			3	2	1	3 (3)
Road traffic accident			2	1	1	2 (2)
Other and unspecified external cause of death		2			2	2 (2)
Assault			1		1	1 (1)
**Non–communicable:**		**2**	**5**	**2**	**5**	**7 (6)**
Acute abdomen		1	1	1	1	2 (2)
Asthma			2		2	2 (2)
Epilepsy		1			1	1 (1)
Severe malnutrition			1	1		1 (1)
Severe anemia			1		1	1 (1)
Total number (%)	23 (21)	33 (30)	54 (49)	59 (54)	51 (46)	110 (110)

*Deaths due to congenital malformations include conditions that have their origin in the perinatal period even though death or morbidity occurs later [34].

**Table 5:** Frequencies of responses to new Verbal Autopsy indicators on circumstances of mortality

	Age group			
	**Neonate (<28 days)**	**Infant (1–11 months)**	**Under 5 years (1–4 years)**	**Total responses to new Verbal Autopsy indicators on circumstances of mortality, n (%)**
**Recognition:**				
Doubts about the need for care	2		3	5 (3)
Use of traditional medicine	3	8	8	19 (13)
**Access:**				
>2 hours to hospital/health facility				
Overall costs prohibitive	3	7	10	20 (14)
Did not use mobile phone	13	12	25	50 (34)
Did not travel to hospital/ health facility	13	9	20	42 (29)
Did not use motorised transport*		5	5	10 (7)
**Quality of care:**				
Problems with admission*				
Problems with treatment*				
Problems with medications*				
Total number of deaths, n (%)	23 (21)	33 (30)	54 (49)	

*The denominator is the number of respondents who reported traveling to hospital/health facility. Respondents were able to indicate more than one 'circumstance of mortality' indicator for each death reported.

After presenting the VA data, we invited participants to share their knowledge and experiences in an open discussion. Participants were prompted to share views on symptoms, modern and traditional therapies, health service responses, and what happens in the village in acute situations. Issues that arose were recorded on a flip chart that was visible to all participants. When a sufficient amount of discussion had occurred in the time that was available, and no new issues were identified, the facilitator (co–author SN) summarized the discussion and checked the list with participants for completeness.

### Collective analyses: ranking and diagramming

**Figure 4:**
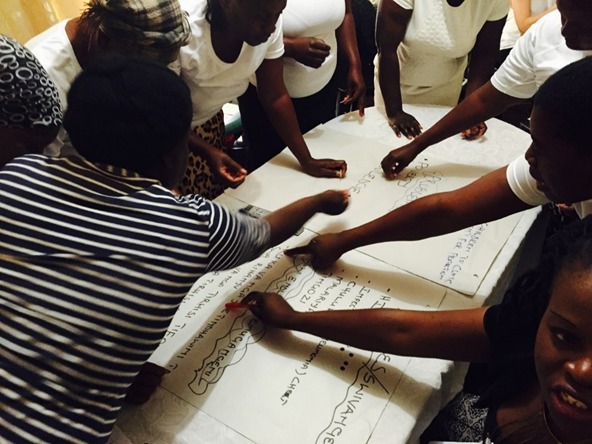
Systematising subjective perspectives – ranking.

**Figure 5:**
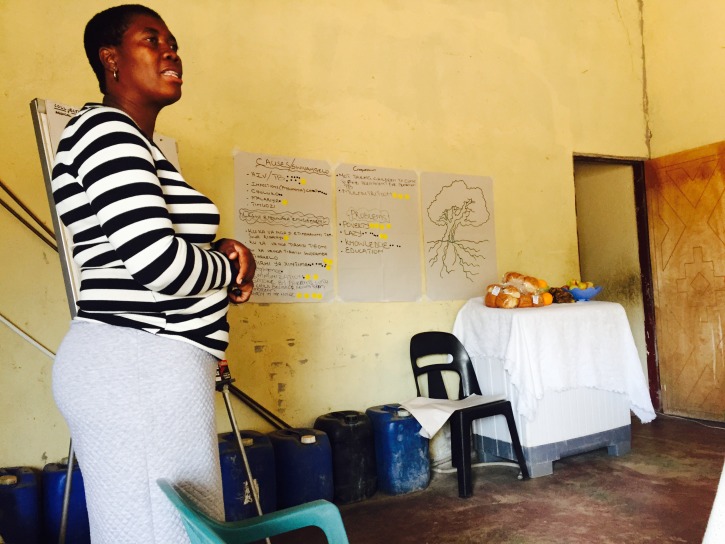
Validating by consensus – diagramming.

We then undertook a process to systematize individual views and experiences into shared accounts using ranking and diagramming. For the ranking, the flip chart with the initial long list was put on a table in the center of the group, and participants were invited to interrogate it. Participants were given adhesive markers to nominate issues they considered to have the highest priority (**Figure 4**). Two rounds of ranking were conducted to ensure the issues were re–visited and re–checked and to validate the list before recording the flip chart in a photograph and closing the meeting. In the subsequent meetings (meeting three) we used diagramming to revisit the ordered list. We adopted a ‘problem tree’ diagram to organize issues identified into proximate determinants, mid–level systems factors, and social and structural causes of under–5 mortality [30] (**Figure 5**).

**Figure 6:**
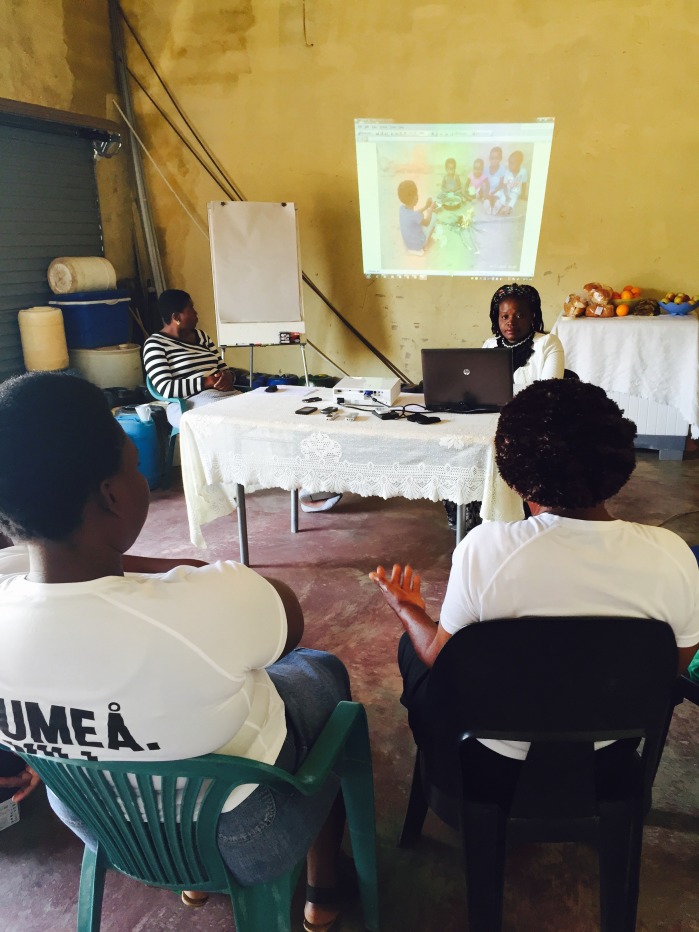
Participatory photography.

One discussion group used a visual participatory technique called Photovoice to explore the use of contemporary methods employing mobile and digital technologies. We selected the remotest, all–female discussion group (Group C) for the Photovoice method. Participants in Group C were provided with digital cameras to take photographs of their physical environments as a further input to the discussions [36,37]. We provided basic training on photography, explained why and how to secure release permissions from subjects of photographs, and provided consent forms for permission releases. In the subsequent weekly meetings, we projected the photographs taken by group members during the discussions. Photographers were invited to describe and explain their images and the group considered the issues they represented as additional inputs to the discussion (**Figure 6**).

### Priority setting

In the final meetings on under–5 mortality, a summary of the process was fed back to each group to verify content and meaning, upon which discussions were held about actions to address the issues identified. This involved re–visiting and re–checking the outputs of the prior process, and moving from causes and contributory factors toward remedial actions. We used ranking to identify priorities for action through which only issues that were nominated by the group were registered. We also indicated that the outputs of the process would be provided to the local health authority.

All discussions were facilitated by a senior qualitative researcher (co–author SN) with knowledge of the local area, assisted by a qualitative field research assistant and co–researchers (co–author LD). The focus group discussion (FGD) method was employed [38] using topic guides to structure the discussions in meetings lasting 90–120 minutes. With separate permissions, the discussions were audio recorded and transcribed verbatim. Transcripts were translated from the local language, xi–Tsonga, into English. SN oversaw and performed transcription and translation with the field assistant. Observational notes were also taken (SN, LD and field assistant) and analyzed.

### Data analysis

The visual and narrative data were thematically analyzed to complement the collective analyses. Thematic analysis was conducted in parallel to, and following completion of, data collection. NVivo Version 10 was used for data entry and management [39]. Transcripts were analyzed based on combined inductive/deductive framework analysis (co–authors OW and LD). This involved a sequence of steps of increasing abstraction from data to findings [40,41]. The transcripts were read several times to familiarize researchers with the main ideas, paying attention to recurring patterns and themes. Initial themes and sub–themes were noted as codes. Transcripts were re–read, re–checking for themes, how themes supported the data and vice versa, identifying relationships within and between themes. This was done iteratively until thematic saturation. The visual data were cataloged and a ‘word cloud’ was generated though which frequencies of terms were graphically represented.

### Ethical considerations

Informed consent was sought from all participants. Participants were provided with information in the local language and contact details for the research team, and given time to consider this before agreeing to be involved. Separate consents were gained for audio recordings. All participants were assured anonymity, and that taking part would have no influence on care available to themselves or their families. Participants were also assured that they were free to leave the process at any time and for any reason. Participants were reimbursed with travel expenses, provided with refreshments in meetings, and given a voucher of ZAR300 (approx. US$ 23) at the end to reimburse for time spent participating and as a token of appreciation. All identifiable data were anonymized. Institutional review boards at the Universities of Aberdeen, Scotland, and Witwatersrand, South Africa, and the provincial health authority in Mpumalanga, reviewed and approved the study protocol.

## RESULTS

The collective analysis is presented below according to two overall categories: a) social and structural root causes and b) contributory mid–level systems factors related to under–5 mortality. The results are illustrated with verbatim quotes and visual Photovoice images from the thematic analysis.

### Social and structural root causes

#### Lack of education, unemployment and poverty

Unemployment and poverty linked to lack of education were identified as root causes of under–5 mortality. Despite free public health care for children under–5, the indirect costs of care seeking were unaffordable for people without regular paid employment. The consequences for service utilization and ultimately health outcomes were clearly stated. Lack of education was referred to as ‘a problem of black people’ more generally, with the need for community health education and activities such as the PAR process noted.

*“…when they say money to take the child to the hospital she would tell you that she doesn’t have money [to travel to the clinic]. And then you find that the child might be unlucky and then die.*” (Woman 3; Village B; Discussion 4)

*“…they are not educated … even this workshop is teaching people about diseases … If there are workshops like this, they are few.”* (Woman 5; Group B; Discussion 4)

*“The bottom line is … our black people need a lot of education.”* (Man 2; Group B; Discussion 4)

#### Lack of clean water

**Figure 7:**
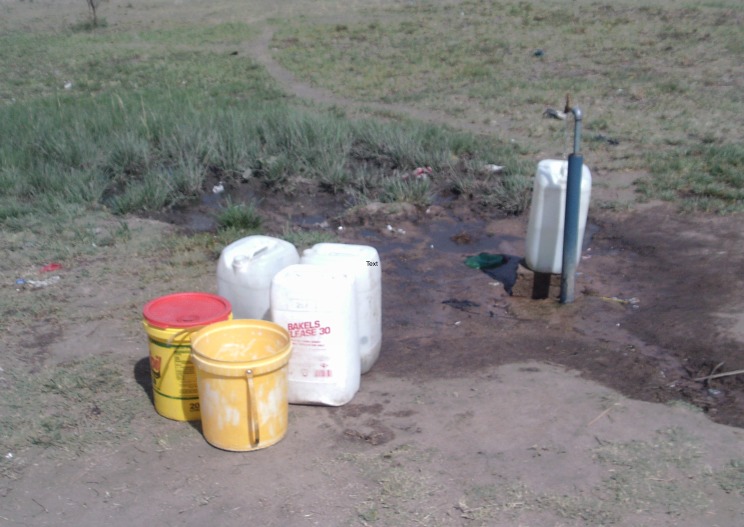
Lack of clean drinking water (Photovoice image).

Lack of clean drinking water was a further fundamental root cause identified. Participants described using rivers and animal water supplies as drinking sources during periods of drought and when pipe–borne water fails (**Figure 7**). Participants also recounted knowledge of children drowning while fetching water in these circumstances.

*“…look carefully… this place is dirty, even our livestock drinks water there, also people drink water there.”* (Woman 5; Group C; Discussion 5)

*“…we are suffering because of water; we go to the streams to dig to get water. Meanwhile the water that we get when we dig it’s not right.”* (Woman 2; Group A; Discussion 9)

#### Unsafe environments and inadequate housing

**Figure 8:**
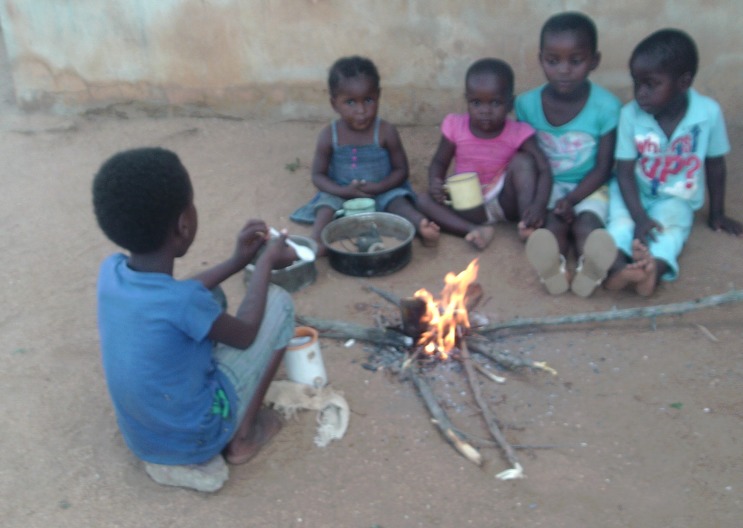
Unsafe domestic environments (Photovoice image).

Unsafe environments and inadequate housing were verified across the groups (**Figure 8**). Further domestic hazards related to water were noted, such as drowning in water storage cans and ingestion of chemicals mistaken for water. Road traffic accidents and sexual assaults were also noted.

*“That child drowned in the water while there are people in the house, they were at home.”* (Woman 5; Village B; Discussion 4)

*“If I have bought 2 litres of paraffin, I have to hide it… the child was thirsty and then he took that 2 litre and drank it.”* (Woman 6; Group C; Discussion 8)

*“…The child can walk into the bush and find snakes and cruel people … there are lots of people in the bush that might rape the child.”* (Man 1; Group C; Discussion 8)

**Figure 9:**
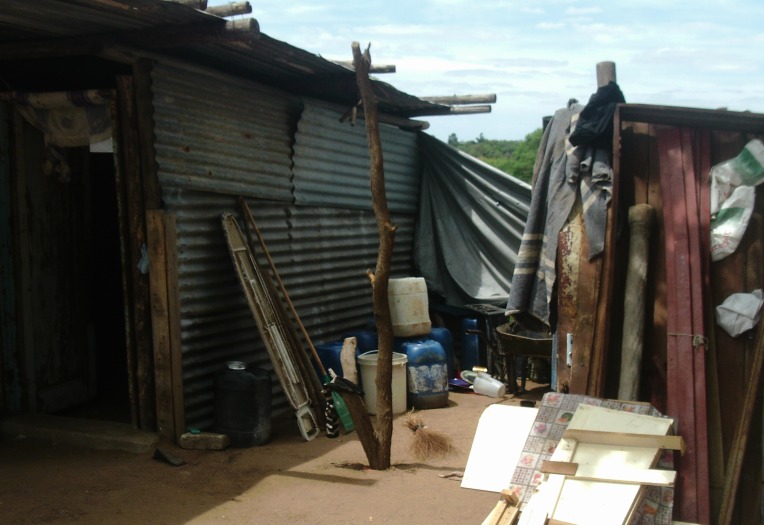
Overcrowding and poor housing (Photovoice image).

Furthermore, inadequate housing and overcrowding were reported to result in children contracting infectious conditions (**Figure 9**).

*“…the cold was coming into the house until my child had pneumonia.”* (Woman 4; Group B; Discussion 4)

#### Malnutrition

Malnutrition was identified as a common cause of ill–health and death in younger children. Malnutrition was also linked to poverty and unemployment, with reference to the inability of parents to purchase nutritious food.

*“… young children died because of hunger.”* (Woman 1; Group B; Discussion 4)

*“… how would I buy milk … the father of my child is not working.”* (Woman 1; Group A; Discussion 6).

#### Perceived parental neglect

The neglect of infants, particularly by young, single mothers was repeatedly noted in the discussions. Specifically, views on the misuse of child support grants (CSGs) were recounted in detail. Discussions also centered on how grandmothers are left with children whose mothers are seeking employment or engaging in vices. Grandmothers were described as unable to properly care for under–5s, further compounding exposure to risk.

*“*[Single mothers] *receive R330 Child Support Grant … She takes that money and uses it or spend it on alcohol, buys pants and cool drink *[participants laugh].*”* (Woman 1; Group B; Discussion 4)

*“Children … can get infections because the way a young person and a granny nurse the child it’s not the same…”* (Woman 1; Group A; Discussion 6)

Lack of recognition of the severity of symptoms on the part of parents (generally young, single mothers) was also identified and related to incomplete health education and limited understandings of health protection and promotion. According to participants, this resulted in the worsening of child health and sometimes death.

*“Another thing is negligence, you find that a child has started diarrhoea and then the parent say it’s nothing and it will pass. And then when the child is too weak it’s when they try the hospital whereas it’s already late.”* (Woman 5; Group C; Discussion 5)

#### Traditional medicines, witchcraft

Participants described how some traditional treatments are harmful to children and delay presentation in clinics and hospitals. However others expressed views that certain illnesses are only curable with traditional medicines. Although there were some differences in views on the use of traditional medicine within the groups, a pervasive lack of faith in modern medicine was identified as a strong influence on its use.

*“…they take rat’s faeces and grind it and put it on the child’s belly button, do you see that I am killing the child when I do that?”* (Woman 5; Group C; Discussion 8)

*“… when a child is sick… just take what we give *[traditional medicine]*… you don’t go to hospitals.”* (Man 2; Group B; Discussion 4)

Witchcraft was also identified as a deterrent to care seeking. Participants recounted how mothers believe that illnesses in children are due to spells cast by neighbors and so conceal and/or do not act on signs and symptoms.

*“There is lots of witchcraft and we don’t trust one another… she won’t help you with anything, which is why there is lots of death because I hide from my neighbour that I have a child who is sick…”* (Woman 2; Group A; Discussion 6)

### Contributory mid–level systems factors

#### Unavailable emergency transport

An unreliable ambulance service was identified as a major problem linked to adverse outcomes. Ambulance delays for several hours after being called were described. Participants reported how unavailable emergency transport results in worsening of children’s conditions and, on occasion, death in the acute situation.

*“…when you call the ambulance it doesn’t come from [hospital 30 km away] but from [hospital 150 km away]. Even the time my child was sick I called an ambulance and they told me that they are busy … I asked my sister to take my child to the hospital because I saw that she would die.”* (Woman 2; Group B; Discussion 4)

#### Delays in facilities

Delays in health facilities was a further issue identified. Specific issues included: overcrowded clinics, long queues and waiting times, and long breaks taken by health workers. Participants described whole days spent waiting and knowledge of situations where children had died while waiting to be seen.

*“When you get to the clinic you have to queue … even when they see that the child is very sick they don’t help you immediately … the child dies because of queuing.”* (Woman 2; Group A; Discussion 9)

#### Poor quality care

Poor quality of care, particularly care provided by nurses, was ascertained as a major contributory factor across the groups. Specific issues included lack of respect for patients and disclosures of HIV status. Participants noted how fear of status disclosure leads to avoidance of ANC, in turn increasing risks to new–borns.

*“If there was confidentiality, people would …go to the clinic when they are pregnant… they are scared to go to the antenatal clinic.”* (Woman 3; Group B; Discussion 7)

*“She never went for antenatal clinic … when she gave birth the child was born infected with HIV.”* (Woman 2; Group A; Discussion 6)

Accounts of nurses treating patients poorly, giving preferential treatment to friends and relatives, not performing triage, and lack of a sense of urgency in acute situations were also verified. Again, fear and avoidance of health services were corroborated among participants.

*“She’s scared to take the child to the hospital because…they are not treating us well.”* (Woman 2; Group A; Discussion 6)

There was some acknowledgment of broader system influences on provider behaviors. Participants noted that health workers are overworked and clinics understaffed. Several also shared knowledge and experience of good nurses.

*“*[Nurses] *are tired because they work with many people…”* (Man 1; Group A; Discussion 6)

#### Lack of medicines

Lack of medicines was a further factor identified that discouraged parents from taking children to clinics. Nurses taking medicines for personal use and further depleting supplies was also noted between the groups.

*“…make sure that the nurses don’t take the medication….that is why we don’t get it sometimes.”* (Woman 3; Group B; Discussion 7)

*“... the child might die because I went where I was supposed to get help but they tell me that there are no medications.”* (Woman 1; Group C; Discussion 8)

**Figure 10:**
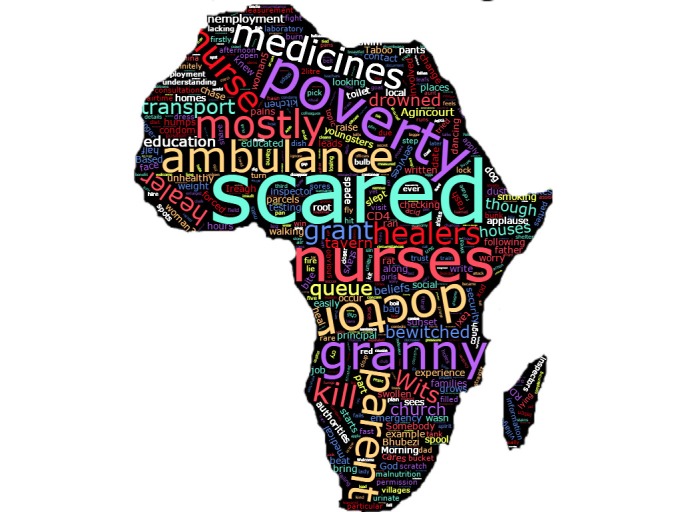
Word cloud of discussion narratives on causes and contributors of under–5 mortality.

The word cloud illustrates the frequency of references to fear of negative consequences from interacting with the health system, quality of care from nurses, transport problems, the effects of poverty, and perceived neglect of children by their parents (**Figure 10**).

### Priority setting

Participants identified areas for action to address the issues identified. These were validated via ranking and arranged according to the two overall categories – “social and structural” and “mid–level systems” priorities (**Table 6**).

**Table 6:** Collective analysis on causes, contributors and priority actions to reduce under–5 mortality

Causes/contributors	Actions
**Social and structural root causes:**	
Lack of education, unemployment and poverty	Generate employment; increase social amenities related to primary and secondary education
Lack of clean water	Provide access to clean water
Unsafe environments/ inadequate housing	Increase social amenities related to road safety
Malnutrition	Implement community based health promotion
Parental neglect	Health education and health promotion campaigns (eg, through PAR process)
Traditional medicine and witchcraft	Encourage medical and traditional healers to work together
**Mid–level contributory systems factors:**	
Transport problems	Build and expand clinics
Delays in facilities	Employ more health workers
Poor quality care	Improve attitudes toward patients; ensure confidentiality; monitor health workers
Lack of medicines	Increase medicines and supplies

#### Social and structural priorities

Considering the influence of poverty – on housing, nutrition, care–seeking, access to clean water, and the combined effects of these on under–5 mortality – participants identified employment as a priority area for action. Participants described how this would empower people to generate income, thereby enabling family health promotion.

*“…they fall pregnant just because they want the money for Child Support Grant. And that grant is little, so I think they can be helped by employment. So everyone could work and end poverty.”* (Woman 2; Group A; Discussion 6)

Expanding health promotion and health education were also prioritized, and approaches to achieve this, for example via media campaigns and local radio were suggested. Participants also stated that the PAR process itself could serve as a vehicle for health education and promotion.

*“… the government needs to help us to broadcast everything that we talk about on television, radio and teach parents so they can be responsible for their children.”* (Woman 4; Group B; Discussion 4)

### Mid–level systems priorities

The need for basic functionality in clinics was highlighted, with the respecting of patient confidentiality around HIV status in ANC identified as a primary priority within this. In addition, the expansion and/or building of more clinics and encouraging medical and traditional healers to work together were further priority areas defined.

*“…at the clinic they have to teach themselves to have confidentiality.”* (Woman 3; Group B; Discussion 7)

*“They have to extend that clinic because there are many people who use that clinic and that clinic is small. People wait outside.”* (Woman 2; Group B: Discussion 8]

*“… make traditional healers and western doctors work together because even what others do it’s working and also the doctors what they do it’s working. Not to say: ‘don’t use other things’ when you use [traditional] treatment … it’s working and most people do use it.”* (Man 1; Group A; Discussion 6)

## DISCUSSION

We initiated a PAR process to gain local knowledge and prioritize actions with communities in an established HDSS. This section reflects on these aims, considers the findings, and discussed implications for further application of the method.

### Substantive findings

Poverty, unemployment, inadequate housing, unsafe environments and shortages of clean water were clearly identified as fundamental root causes of under–5 mortality. Considering the high proportion of under–5 deaths due to infectious diseases, it is reasonable to assert that critical risk is introduced from the environmental conditions identified. It is also noteworthy that the priorities identified to respond to these issues (improving employment, providing clean water and improving road safety) are beyond the remit of the Department of Health. We re–visit this point in the methodological reflections below.

Despite the clear acknowledgment of the influence of social conditions on under–5 mortality, marked criticism was expressed toward young single mothers for the neglect of children and infants and the misuse of the CSG. CSG is a social protection intervention which covers 12 million children in South Africa. It is available to primary carers earning below a means–tested benchmark. At the time of data collection, the CSG provided ZAR330 (approx. US$ 25) per month per child [42,43]. Studies have linked the scheme to increased quality of life, better access to services, and reductions in adolescent pregnancy with little perverse incentive [44,45]. Others however assert that such schemes may have limited benefits for those already engaged in risky behaviors, noting influences other than material deprivation on loss of responsibility [46–48]. The findings highlight both the limitations on parents’ abilities to protect and promote family health, and local views on individuals’ accountability for these situations.

A series of mid–level health systems factors were also identified as contributory to adverse outcomes. Lack of confidentiality around HIV status, disrespect and abuse of patients, and misuse of medications on the part of clinic nurses were identified as deeply problematic. Poorly staffed and equipped clinics, long waiting times and overcrowded facilities were also ranked as key influences. The expressions of fear and avoidance of services due to poor quality care supports ideas of how repeated interactions between providers and users of services shape, and are shaped by, social norms of eligibility for services [49]. Again, the blame directed toward clinic nurses was expressed with simultaneous acknowledgment of wider systems constraints.

The health system in South Africa faces far–reaching challenges. Service provision is distinctly two–tiered with over 70% of physicians working in the private health sector catering for an affluent 16% of the population [19,50,51]. The public arm of the system is described as fragmented and dysfunctional, with systemic failures in leadership, stewardship and implementation [52]. Clinics are acutely resource–constrained, and staff are chronically over–worked as a result [19,25]. The two–tier system was explicitly acknowledged in the recent White Paper for NHI in a bold move toward universal access to a basic minimum package of essential health care services available on the basis of need and without financial hardship [22].

The White Paper commits to substantial reorganization of the health system in a phased 14–year implementation comprised of three phases: (1) strengthening quality of care; (2) registering the population, distributing NHI cards and procuring services and; (3) assessing functionality and sustainability via audit, demographic and epidemiological population profiling [22]. A policy of PHC Re–engineering was introduced in 2011 to support the first phase. The policy focuses on improving connections between services and communities through community (ward) based outreach teams, health promotion in schools, and scaled up attention to maternal and child health [53]. PHC Re–engineering is an opportunity to develop the relationships between health authorities and communities to inform decentralized PHC, to foster more positive care contexts and interactions. The recommendations for action on enlarging community engagement for health promotion and education are consistent with the aims of PHC Re–engineering. Participants also noted that the PAR process was a suitable means for expanded engagement.

The work developed an initial understanding of context and common conditions. Priority actions were specified, fed back to the provincial directorate [54], and subsequently a feedback forum was held between the Department of Health and participants in the Agincourt HDSS [55,56]. Within the time and resources, it was not possible to develop the process into taking action, and reflecting on and learning from action, and so PAR was not fully achieved. Through the activities undertaken however, willingness and commitment were expressed by participants and health authorities to continue the process into taking action, and reflecting and learning from this action [57]. Extending the process will add a crucial link to understand how change occurs in health systems, by which means, for whom, and on the role of evidence in the process. We consider these points further below.

### Methodological reflections

#### Participation for whom?

As the process continues, who participates is a key consideration. In the introductory work, we sought to convene village–based discussion groups with shared social and health conditions. We developed a geographically defined group as residents of a specific area and varied the constituency of the groups to be more or less homogenous. There were no overt differences observed in the dynamics between the groups in the discussions on under–5 mortality, underscoring ideas that no group is entirely homogenous [30].

A prominent finding common to all the discussions was the blame and negativity expressed toward clinic nurses and young single mothers – two females at the care interface who arguably have limited control over the wider conditions identified. Despite several participants being young mothers themselves, the blame and negativity was clearly directed toward the behaviors of “others”. Similar dynamics have been observed in participatory research elsewhere, whereby initially blaming views, over time, gave way to sophisticated multi–level interpretations of complex problems [58].

The importance of maintaining links with the groups convened to date as well as expanding the process to include the perspectives of child–headed households, young mothers and clinic nurses will be central to considerations of whose voices count in the process in future. Close attention is also required on whether and how views around blame, responsibility and accountability develop in the longer term.

#### By which means?

The importance of actively disrupting hierarchies between researchers and participants is a further feature to be developed in future application of the method [59]. Promoting and building participants’ capacities as co–researchers, decision makers, action takers and influencers of institutional decision makers is necessary for communities to own the process and the development of the process.

In this research, we made efforts to foster a sense of control by encouraging participants to determine how the topics were selected and framed, and which issues would be referred to the health authority. As the process continues, increasing participant control over the design, process, choice of topics, how outputs are discussed, communicated, acted on and learned from will help build roles related to community ownership and capacity [30,60,61].

Photovoice is a capacity building process in which participants gain skills that can be used to earn income [36]. In this study, Photovoice fostered participation, was a low–cost means of generating powerful evidence and strengthened the process as a whole. The use of Photovoice to investigate quality of care in health facilities however may be more contentious, and is an area that requires further consideration with providers and participants.

Photovoice was one of a range of analytical approaches employed in the initialized process. The thematic analysis was performed to fully interrogate the data collected and illustrate the collective analysis. It also identified the paradoxical blame. The different approaches have supported one another, triangulated findings, and helped to identify relevant divergences.

#### How change occurs in health systems

Connecting the process to the means for action is critical [61,62]. The initialized process was developed in close collaboration with the Public Engagement Office of the Agincourt HDSS, a group that has strong and sustained links with communities and health authorities at different levels. Through these links it was possible to connect communities, health authorities and researchers to develop and address common research questions and adopt roles as co–researchers.

We plan to extend what has been achieved to date into an ongoing cycle of reflection and action inclusive of district, sub–district and clinic level to facilitate and increase the co–production of policy–relevant evidence to understand and respond to priorities. The importance of engagement with sectors adjacent to health – such as labor, housing, and sanitation as well as the local authority – will help to foster an integrated approach to using the information generated to achieve change in health systems [63].

#### The role of evidence

This work adds to the literature on researching the social determinants of mortality in HDSSs. Verbal and social autopsy surveys have collected data on social determinants of child mortality in the INDEPTH Network (International Network for the Demographic Evaluation of Populations and Their Health), HDSSs [64,65] and at district and national level [66,67]. The latest version of the WHO VA tool also includes questions on circumstances of mortality that have been developed as part of, and used within, the current project [35].

These efforts reflect the need for methods to provide better understandings of the contextual determinants of mortality in low income settings. Providing PAR methods and data to HDSSs in other settings is also prioritized to promote further application of the method to provide complementary perspectives gained from local knowledge and oriented toward action [68,69]. This is relevant in South Africa as the government consolidates and expands national HDSS infrastructure as a means to inform public policy in the country [70].

## CONCLUSIONS

*“Health systems can, in the way they function, strengthen the capabilities of individuals and social groups, for example by including opportunities for people to participate in planning services, from individual care plans to community health interventions.”* (page 11 in [30])

We initiated a PAR process to gain local knowledge and prioritize actions. This study provides evidence that consulting communities provides rich and textured information on the social and health systems dimensions of avoidable mortality. Widespread poverty, unemployment, poor housing and inadequate water were repeatedly identified as direct causes of death within and between the village–based discussion groups. Health systems factors were also clearly identified as contributing to mortality.

The process also helped to establish a commitment to partnerships between communities, health authorities and researchers. The study provided a basis from which to gain support to develop fuller forms of participatory research in this setting. The next steps are under way to build deeper involvement of participants in the process, expand to include the perspectives of those most marginalized, and to further develop engagement with health systems stakeholders to enable action, and learning from action. In combination with routine HDSS, the use of PAR to elicit local knowledge on health problems has the potential to connect communities, researchers and health authorities to develop robust evidence for service delivery, policy and planning.
